# The Potential Safe Antifibrotic Effect of Stem Cell Conditioned Medium and Nilotinib Combined Therapy by Selective Elimination of Rat Activated HSCs

**DOI:** 10.1155/2021/6678913

**Published:** 2021-03-28

**Authors:** Ahmed Nabil, Koichiro Uto, Faten Zahran, Reham Soliman, Ayman A. Hassan, Mohamed M. Elshemy, Islam S. Ali, Mitsuhiro Ebara, Gamal Shiha

**Affiliations:** ^1^Research Center for Functional Materials, National Institute for Materials Science (NIMS), 1-1Namiki, Tsukuba, Ibaraki 305-0044, Japan; ^2^Biotechnology and Life Sciences Department, Faculty of Postgraduate Studies for Advanced Sciences (PSAS), Beni-Suef University, Beni-Suef, Egypt; ^3^Egyptian Liver Research Institute and Hospital (ELRIAH), Sherbin, El Mansoura, Egypt; ^4^Biochemistry Department, Faculty of Science, Zagazig University, Egypt; ^5^Tropical Medicine Department, Faculty of Medicine, Port Said University, Egypt; ^6^Faculty of Science, Menoufia University, Menoufia, Egypt; ^7^Delta University for Science and Technology, Egypt; ^8^Graduate School of Pure and Applied Sciences, University of Tsukuba, 1-1-1 Tennodai, Tsukuba, Ibaraki 305-8577, Japan; ^9^Graduate School of Industrial Science and Technology, Tokyo University of Science, 6-3-1 Niijuku, Katsushika-ku, Tokyo 125-8585, Japan; ^10^Hepatology and Gastroenterology Unit, Internal Medicine Department, Faculty of Medicine, Mansoura University, Egypt

## Abstract

Hepatic fibrosis is a progressive disease with serious clinical complications that arise from abnormal propagation and activation of multiple inflammatory pathways. Nilotinib is an oral tyrosine kinase inhibitor with antifibrotic activity. Mesenchymal stem cells (MSCs) are blank cells and can differentiate into specific cell types. They have the potential to repair and regenerate cells. MSCs have a special paracrine fashion where they produce special exosomes, microvesicles, and cytokines like IL-6, transforming growth factor-beta (TGF-*β*), and HGF as well as hepatic stellate cell suppressors. This paracrine fashion can decrease collagen deposition, enhance antifibrotic, anti-inflammatory, and angiogenic activity in vitro and in vivo. In our study, the rat's hepatic stellate cells (HSCs) in addition to different normal cell lines were treated with Nilotinib alone and in combination with liver mesenchymal stem cells conditioned medium (LMSCs-CM) for 24 h. Mono and combined therapy antifibrotic and cytotoxicity effects were evaluated using different parameters including *α*-SMA, cytochrome c, P53 expression, collagen deposition, DNA content, oxidative stress parameters, cell viability, and apoptosis by flow cytometry analysis. Our results showed that Nilotinib and LMSCs-CM in combination had a significantly potent antifibrotic and anti-inflammatory effect on activated hepatic stellate cells than Nilotinib alone; otherwise, this combination showed the best safety with minimal cytotoxicity on different normal cell lines.

## 1. Introduction

Liver fibrosis is a common pathological outcome of different chronic liver diseases, like hepatitis B and C virus infection, alcoholic liver disease, and nonalcoholic steatohepatitis (NASH) that may cause cirrhosis and liver tumors. Extracellular matrix proteins (ECMs), including collagen type I, are the key event in hepatic fibrous scar formation. Mainly, myofibroblast activation is responsible for this fibrous scar formation and the increased deposition of ECMs [1]. Moreover, activated hepatic stellate cells (HSCs) and portal fibroblasts are classified as collagen-generating cells in the damaged liver. HSCs are activated through fibrogenic cytokines like TGF-*β*1 and angiotensin II. Thus, reversal or regression of fibrosis depends on the inhibition of fibrogenic cell accumulation and preventing ECM deposition [2].

Tyrosine kinase inhibitors (TKIs) compete with adenosine triphosphate (ATP) for the binding sites and then decrease TK phosphorylation hindering tumor cell proliferation [3]. Nilotinib is a second-generation TKI that plays a role in the regression of hepatic fibrosis; it reduces collagen formation and enhances liver fibrogenolysis [4].

Mesenchymal stem cells (MSCs) were obtained from various tissues like bone marrow, adipose tissue, and liver. MSCs had multipotent differentiation, immunomodulatory characteristics, and self-renewal capacity, allow the replacement of injured hepatocytes, enhance hepatocyte renewal, and suppress HSCs activation or stimulate their apoptosis. Thus, MSCs are considered an appealing therapeutic method. [5]. Moreover, MSCs have a paracrine fashion where they form distinct exosomes and microvesicles, in addition to large amounts of antiapoptotic agents like interleukin (IL-6) and insulin growth promoter (IGF-I) and anti-inflammatory agents like interleukin 1 receptor antagonist (IL-1Ra) [6]. MSC conditioned medium (MSC-CM) includes special exosomes, growth agents, and cytokines that have an anti-inflammatory impact, inverse hepatic fibrotic state, and improve liver recovery [7].

Liver mesenchymal stem cells (LMSCs) create a high level of proangiogenic, anti-inflammatory, and antiapoptotic cytokines. Furthermore, LMSCs express albumin, CD26, and CK8 giving engagement to hepatocyte-like cell transdifferentiation, gaining various hepatic functions like cytochrome P450, albumin, and urea formation [8]. Additionally, LMSCs-conditioned medium (LMSC-CM) contains high levels of IL-10 and tumor necrosis factor- (TNF-) *α* that decrease HSCs and collagen-I deposition, as well as IFN-*γ* production, to provoke antifibrotic activity [9]. Thus, LMSC-CM is a hopeful therapeutic tool for different hepatic diseases. In our study, we presented new intuitions regarding the antifibrotic effect of Nilotinib alone or combined with conditioned media in addition to cytotoxicity evaluation.

## 2. Materials and Methods

### 2.1. Laboratory Animal

Ten male (Sprague-Dawley) rats, weighing 450 to 700 g (20-30 weeks), were used for HSCs isolation (heavy and aged rats were used as it had a high content of cells). Liver MSCs were isolated from fifteen female Wistar rats weighing 60 g to 80 g (3-4 weeks). The animals were kept under a light-dark cycle (12 : 12 h), 21 ± 2°C, and 50 ± 5% relative humidity with food and water ad libitum. This research was conducted at Liver Research Institute and Hospital (ELRIAH) (Sherbin-El Mansoura-Egypt) in the duration between March 2018 and June 2019. Ethical rules relating to laboratory animals' trials and treatment were agreed upon and followed under the ELRIAH Experimental Animals Ethical Committee (No. ELRIAH/EAEC/CRL/TCU/3/02, 2018). Rats were brought from the Medical Experimental Research Center- (MERC-) El Mansoura-Egypt.

### 2.2. Isolation of MSCs and Conditioned Media Production

Rats were exposed to overdoses of thiopental anesthesia according to their body weight followed by cervical dislocation; then, LMSCs were isolated using the method described previously [10, 11]. After 3 passages, cells were cultivated (10000 cells/cm^2^) and preserved in complete culture media (DMEM supplemented with 10% FBS, 1% antibiotic-antimycotic, 1% nonessential amino acids, and 1% L-glutamine) for a day. The LMSCs were washed 3 times using PBS and preserved for a day in serum-free basal media. Then, the supernatant was obtained for centrifugation (1500 × g for 10 min) and concentrated 50-fold using ultrafiltration units (5 kDa cut-off) (Millipore, Bedford, MA, USA). The obtained MSC-CM was preserved at -80°C until use.

### 2.3. Hepatic Stellate Cell Preparation and Activation

Rats were exposed to overdoses of thiopental anesthesia according to their body weight followed by bilateral thoracotomy; then, rats' primary HSCs were isolated using digestive enzymes (collagenase and pronase) followed by cell suspension density gradient centrifugation. HSC cells were cultured in Dulbecco's modified Eagle's medium (DMEM). HSCs kept in the culture at 37°C, 5% CO2, and 100% humidity; cells become active after seven days of initial culture [12].

### 2.4. MTT Assay (Viability Test)

HSCs, LMSCs, WI-38 (human lung fibroblast), MDBK (cow normal kidney), and CHO-K1 (normal ovary of an adult Chinese hamster) were cultured in DMEM at 37°C and CO_2_ 5% with FBS 10% and penicillin 100 U/mL. Evaluation of viability using the MTT Kit [13] which assesses cell metabolic activity depends on the reduction of MTT dye to violet formazan crystals by succinate dehydrogenase inside living cell mitochondria. The cells were cultured in a plate (96-well) (Greiner, Frickenhausen, Germany) (6000 cells/well) and then preserved at 37°C and 5% CO_2_ for a day. The medium was discarded, and various concentrations (10-50 *μ*M) of Nilotinib (formerly AMN107; Tasigna®, Novartis, Basel, Switzerland) were dissolved in DMSO and added in the wells. Each concentration of Nilotinib was incubated twice, one with ordinary serum-free media and the other with liver mesenchymal stem cell conditioned media. After another incubation with the same conditions, the medium was discarded, and 100 *μ*L MTT (2 mg/mL) was added and preserved 3 h at 37°C. The produced purple formazan crystals were suspended in 50 *μ*L of DMSO. Then, the plate was incubated (15 min, at 37°C, 5% CO_2_). Cell viability was calculated at 570 nm using an Infinite® 200 PRO plate reader. DMSO was used as a solvent with a concentration of less than 0.2%. The half-maximal inhibitory concentration (IC_50_) and selectivity index (SI) were calculated. All in vitro tests were performed in triplicate.

### 2.5. Apoptosis Flow Cytometric Analysis

HSCs were treated with Nilotinib alone and Nilotinib + MSC-CM at the concentration of IC_50_ for 24 h. The cells were labeled with annexin V–fluorescein isothiocyanate (AV-FITC) apoptosis kit (Miltenyi Biotec GmbH) and propidium iodide (PI) (Miltenyi Biotec GmbH). The cells were washed twice by PBS and fluorescence intensity assayed by MACS Quant Flow Cytometer and Miltenyi Biotec GmbH.

### 2.6. Cytotoxicity Assay Using Trypan Blue

Trypan blue selective staining of dead cells followed by the microscopic investigation is considered a common routine technique to detect cellular viability [14]. An equal volume of HSCs suspended in FBS mixed with Trypan blue solution 0.4% (Sigma). After 5 min, an aliquot of the mixture was checked in a Neubauer chamber at 20°C, and then, viable and nonviable cells within four big squares in the four corners were counted through phase-contrast microscopy [15]. Cell viability (in %) was evaluated as follows:

(1) $$\begin{array}{c} \left[\frac{\text{Total viable cells }\left(\text{unstained}\right)}{\text{total cells }\left(\text{stained and unstained}\right)}\right]\times 100. \end{array}$$

### 2.7. Quantification of Collagen Deposition

Collagen was quantified using a Sircol red assay kit (Biocolor, Belfast, Northern Ireland) as follows. HSCs treated with Nilotinib with or without liver MSC-CM at the concentration of IC_50_ were prepared. The medium was removed; 0.5 M cold acetic acid (1 mL/well) was added to harvest the collagen extract. 200 *μ*L of the cold collagen concentration and isolation solution that contained polyethylene glycol in Tris-HCl pH 7.6 was added and mixed well with the collagen extract. This mix was incubated overnight at 4°C. Tubes were centrifuged at 12,000 r.p.m. for 10 minutes; then, very carefully, the supernatant was removed, and 1 mL of Sircol dye reagent was added followed by absorbance measurements.

### 2.8. *α*-SMA, P53, and Cytochrome c Western Blotting Analyses

HSCs were treated with Nilotinib alone and in combination with MSC-CM at the concentration of IC_50_, washed with ice-cold PBS, and cells lysed in lysis buffer (Cell Signaling, Beverly, MA) for 20 min, and then centrifuged at 12,000 g (at 4°C, 10 min). Whole-cell protein extract prepared from treated cells. Sodium dodecyl sulfate-polyacrylamide gel electrophoresis was used for protein (30~50 *μ*g/lane) separation; then, proteins were transferred to a polyvinylidene fluoride (PVDF) membrane (Millipore, MA, USA) and determined by the primary anti-*α*-SMA, P53 antibodies, and horseradish peroxidase-conjugated secondary antibodies. Moreover, cytosolic and mitochondrial cytochrome c fractions were separated by cytochrome c releasing apoptosis kit (Abcam, Cambridge, UK), where cells centrifugated and washed using PBS. Cells were resuspended in the cytosol buffer mix containing DTT with protease inhibitor and then homogenized in an ice-cold Dounce tissue grinder. Then, the homogenate was centrifuged (at 700 × g, at 4°C for 10 min). The supernatant was centrifuged at 10,000 g for 30 min to collect the cytosolic fraction. The pellets resuspended in the buffer mix of mitochondrial extraction and kept as a mitochondrial fraction. Both mitochondrial and cytosolic fractions were analyzed using cytochrome c monoclonal antibody [16]. The immune complexes were visualized with an enhanced chemiluminescence kit (Millipore, Bedford, MD, USA) and (*β*-actin) used as an internal control.

### 2.9. Intracellular Reactive Oxygen Species (ROS)

HSCs were treated with Nilotinib alone and in combination with MSC-CM at the concentration of IC_50_ were resuspended in PBS for homogenization on ice followed by centrifugation to remove cellular debris. 50 *μ*L of each sample was added to 50 *μ*L of hydrogen peroxide working solution into the microplate well. The microplate well contents were mixed well and incubated for 30 minutes at 20°C in darkness. The plate absorbance was evaluated using a microplate reader at 550 nm.

### 2.10. DNA Content Measurement

HSCs cells were treated with Nilotinib with or without liver MSC-CM for a day at the concentration of IC_50_. Cells lysed in NaH2PO4/Na2HPO4 50 mmol/L, pH 7.4, NaCl 2 mol/L, and EDTA 2 mmol/L, and the DNA was assessed as described by Labarca and Paigen [17], where all cell lysates were mixed with 0.1 *μ*g/mL bisbenzimide (Hoechst 33258) in 10 mM Tris-HCl, 0.2 M NaCl, and 1 mM ethylenediaminetetraacetic acid (EDTA) (pH 7.5) followed by fluorescence evaluation with excitation at 356 nm and emission at 458 nm.

### 2.11. Statistical Analysis

Descriptive statistics calculated in the form of mean ± standard deviation (SD). The statistical significance was determined by one-way ANOVA and Tukey's post hoc test, and the effect size statistics were assessed by partial eta squared by the SPSS software (version 22). A level of *P* <0.05 was defined as statistically significant.

## 3. Results

### 3.1. Nilotinib and MSC-CM Cytotoxic Activity

To investigate the cytotoxic effect of Nilotinib alone and in combination with MSC-CM on HSCs and normal cell lines, different Nilotinib concentrations (10-50 *μ*M) were used in cell treatment for a day, and their survival rate was estimated using MTT kit. Each concentration was incubated twice, one with ordinary serum-free media and the other with liver mesenchymal stem cell conditioned media. The results showed that Nilotinib alone and in combination with MSC-CM induced HSC death depended on the dose, IC_50_, and SI values calculated as shown in Table 1. Nilotinib in combination with MSC-CM had a small IC_50_ value (10 *μ*M) and high SI (values: 4.5 to >5) compared with Nilotinib alone (IC_50_ = 25 *μ*M and SI = 0.6 to 1.6) which indicated that the combination of Nilotinib + MSC-CM had high potency and selectivity to the activated HSCs with minimal cytotoxicity on the liver, lung, ovary, and kidney cell lines.

### 3.2. Nilotinib and MSC-CM Treatment Effect on HSC Apoptosis

**Figure 1:**
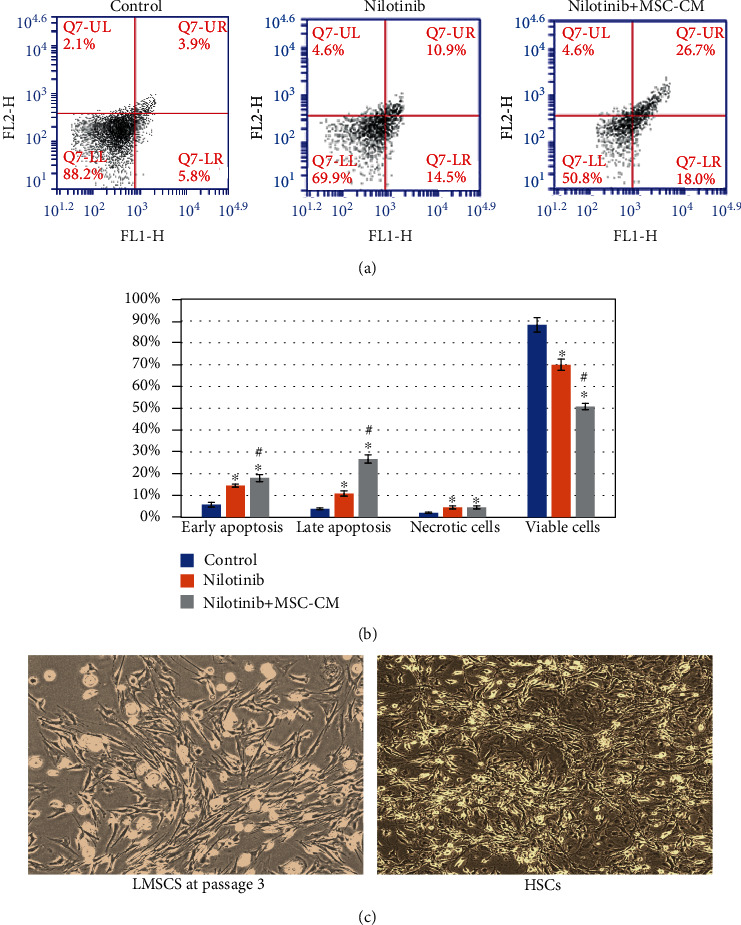
The effect of Nilotinib and MSC-CM treatments for 24 hr on early and late (lower and upper right quadrant, respectively) apoptosis percentage, viable cells in the lower left and necrotic cells in the upper left quadrants (a). Percentage of viable and apoptotic cells (b). Images of HSCs and LMSCs (c). The results showed as mean ± standard deviation, also all samples measured in triplicate. Statistically significant differences indicated as ^∗^*P* < 0.001 versus the control group; #*P* < 0.001 versus the Nilotinib group.

We evaluated the effect of Nilotinib alone and in combination with MSC-CM on HSC apoptosis at the concentration of IC_50_ using annexin V-FITC and PI staining (Figure 1(a)). Results reported a significant decrease in the percentages of viable cells compared with the control group, and both apoptotic and necrotic cell percentages were markedly increased after treatment with Nilotinib alone and in combination with MSC-CM. Treatment with Nilotinib and MSC-CM represented a higher reduction in the percentage of viable cells in addition to a significant increase in late apoptotic cell percentages than Nilotinib alone (Figure 1(b)). Isolated HSC and LMSC images are shown in Figure 1(c).

### 3.3. Effect of Nilotinib and MSC-CM on *α*-SMA, Cytochrome c, and P53 Expression in HSCs

**Figure 2:**
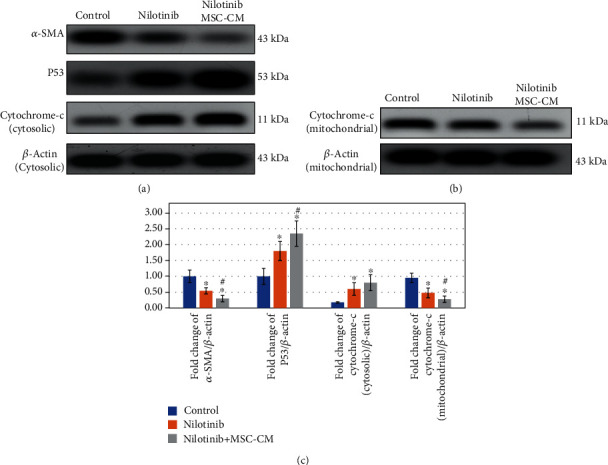
The effect of Nilotinib and MSC-CM on *α*-SMA, cytochrome c, and P53 expression in HSCs. Western blotting assays were carried out to show the expression of *α*-SMA and p53 protein (a) in addition to both cytosolic (a) and mitochondrial (b) cytochrome c expression in HSCs. Western blotting was done by using an anti-*β*-actin antibody to assure an equal load of protein in every lane. Then, bolts were photographed and quantitated for each sample, and the results were from 3 independent experiments. The fold changes of *α*-SMA, cytochrome c, and P53 expression (c). Data are shown as mean ± standard deviation, and statistically marked differences are indicated as ^∗^*P* < 0.001 versus the corresponding control group; #*P* < 0.001 versus the corresponding Nilotinib group.

To evaluate the impact of Nilotinib and MSC-CM treatment on activated HSCs, the intracellular expression of *α*-SMA was examined by western blotting. We observed a significant decrease in *α*-SMA expression, an indicator of HSC activation (Figure 2(a)), after treatment with Nilotinib with or without MSC-CM. Activation of HSCs increased collagen synthesis and deposition, but all treatment regimens had a marked (*P* < 0.001) suppression of collagen deposition in activated HSCs. The protein expression of p53 increased significantly in HSCs after 24 h of treatment with Nilotinib alone and in combination with MSC-CM (Figure 2(a)). Moreover, DNA content in cells decreased significantly (*P* < 0.001) in all treated groups compared with the control group due to DNA damage and p53 protein elevation. The treatment of Nilotinib + MSC-CM had a more significant reduction (*P* < 0.001) in the *α*-SMA expression, collagen deposition, and DNA content and a more significant increase in p53 protein expression compared to Nilotinib alone. Furthermore, the protein expression of (cytosolic) cytochrome c (Figure 2(a)), but not (mitochondrial) cytochrome c (Figure 2(b)), increased significantly after treatment with Nilotinib with or without MSC-CM, but Nilotinib + MSC-CM treatment had a marked elevation in the expression of (cytosolic) cytochrome c, but not (mitochondrial) cytochrome c, compared with monotherapy and vice versa for (mitochondrial) cytochrome c.  Figure 2(c) shows the fold change of *α*-SMA/*β*-actin, P53/*β*-actin, cytochrome c (cytosolic)/*β*-actin, and cytochrome c (mitochondrial)/*β*-actin in HSCs.

### 3.4. Oxidative Stress, Collagen, and DNA Content

This study showed a marked increase in ROS production as well as a decrease in DNA and collagen deposition in all treated HSCs (*P* < 0.001) compared to the control as shown in Table 2. The combination treatment of Nilotinib + MSC-CM revealed a more significant increase (*P* < 0.001) in ROS generation as well as a more significant decrease in DNA content and collagen deposition compared with Nilotinib alone.

### 3.5. Effect of Nilotinib and MSC-CM on Cell Viability

**Figure 3:**
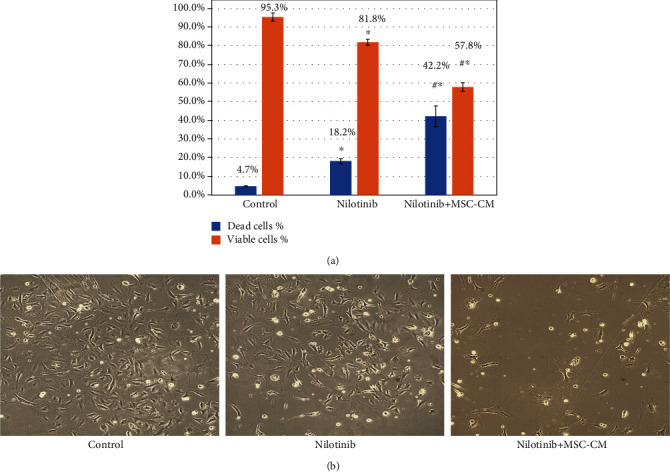
Viable and dead cell percentages of cultured HSCs estimated by Trypan blue stain following 24 h exposure of cells to Nilotinib with or without MSC-CM (a). Morphology of HSCs with different treatments (b). Statistically marked differences indicated as ^∗^*P* < 0.001 against the control group; #*P* < 0.001 against the Nilotinib group.

Viable and dead cell percentages calculated using Trypan blue dye staining. Nilotinib alone or in combination showed a marked growth in dead cells% and marked reduction of viable cell percentage (*P* < 0.001) compared to control. Moreover, Nilotinib and MSC-CM combined treatment showed a more marked increase in the percentage of dead cells and a marked reduction in the percentage of viable cells (*P* < 0.001) compared with Nilotinib alone (Figure 3(a)). Effect of Nilotinib alone and combined with MSC-CM on HSCs morphology showed in (Figure 3(b)).

## 4. Discussion

**Figure 4:**
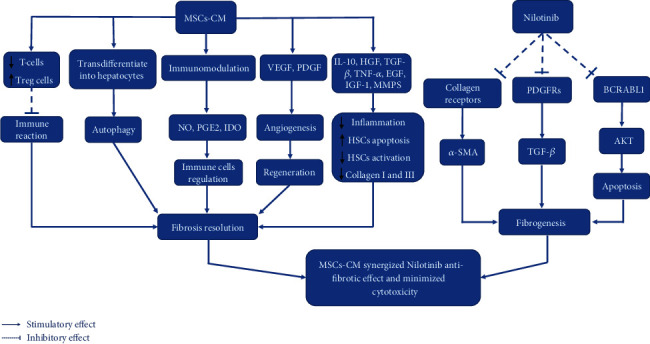
The proposed mechanism by which MSCs-CM synergized Nilotinib antifibrotic effect and minimized cytotoxicity.

In the current study, we examined the antifibrotic impact and safety measurements of Nilotinib + MSC-CM on activated HSCs compared with Nilotinib alone. The current study is the first study to evaluate the antifibrotic efficacy and safety of this combination in vitro.  Figure 4 shows the possible synergism between Nilotinib and MSC-CM to perform more powerful antifibrotic action on activated rat HSCs with minimal cytotoxic impact.

HSCs, the main liver cells associated with the ECM proteins accumulation, are incited by various cytokines like platelet-derived growth factor (PDGF), TGF-*β*, TNF-*α*, IGF-I, endothelin-1 (ET-1), and ROS [18]. Previous studies demonstrated the role of HSC apoptosis during liver fibrosis regression [19]. Consequently, selective elimination of activated HSCs by programmed cell death initiation may represent a therapeutic approach in liver fibrosis treatment. Parallel to our results [20] reported that Nilotinib induces apoptosis of HSCs, DNA fragmentation, and elevation of the p53 protein expression. Our results agreed with previous studies which approved that Nilotinib inhibits the activity of TKs like PDGF receptors (PDGFRs) and nonreceptor TKs like Abelson kinase (c-Abl) [21]. As a result, PDGF stimulates fibroblast proliferation, and TGF-*β* stimulates the fibrogenesis process, interfered by c-Abl via a non-Smad mechanism. Moreover, Nilotinib suppresses collagen receptors [22]. Therefore, Nilotinib had an antifibrotic effect via 3 pathways implicated in fibrogenesis.

Furthermore, our data agreed with [23] who reported that Nilotinib hindered proliferation, migration, actin filament production, collagen deposition, and *α*-SMA composition. Moreover, Nilotinib provoked HSC apoptosis, which associated with a reduction in Bcl-2 levels and an increase in p53 levels. Our results were parallel to previous studies by Shaker et al. [24] which showed that Nilotinib treatment resulted in the cleavage of caspases, and large DNA destruction correlated with the increase of p53 protein-induced HSC apoptosis.

On the other hand, our results showed synergism between Nilotinib and MSC-CM by fibrinogenesis decrease and fibrinolysis increase, which executed more effective antifibrotic activity against HSCs than Nilotinib alone (*P* value <0.001). MSC-CM contains special exosomes, hepatocyte growth factor (HGF), vascular endothelial growth factor (VEGF), antiapoptotic factor IL-6, and anti-inflammatory cytokines like IL-10 effective in the treatment of liver fibrosis through inhibiting HSC activation also trigger HSC apoptosis and fibrinolysis. Moreover, MSCs produce matrix metalloproteinases which have a fibrinolytic activity and reduce the ECM [6]. Parallel to our findings, previous results by Yin et al. [25] showed that MSC-CM has supportive roles in tissue repairing and recovery through attenuating inflammation, enhancing proliferation, suppressing apoptosis, and facilitating angiogenesis.

Various in vitro researches confirmed the capability of MSCs to modify HSC activation indirectly by paracrine methods as well as directly through cell-cell connections [26]. Our study agreed with Chen et al. [27] who found that bone marrow MSCs significantly suppress rat HSC proliferation and decrease *α*-SMA expression level and collagen deposition, which promoted the recovery of damaged hepatocytes. Moreover, a previous study by van Poll et al. [28] found that soluble agents discharged by MSCs promote liver improvement in response to intense injury. For instance, human MSCs-CM injection into rats with hepatic injury improves liver function after 24 h, blocks the emission of liver damage biomarkers, results in a 90% decline in apoptotic hepatocellular damage, and a three-fold rise in whole proliferated hepatocytes.

Regarding MSC safety, our results were agreed with Lalu et al. [29] who analyzed MSC clinical safety in 36 studies, and they reported that MSCs had no tumorigenic potential or side effects during treatment. Additionally, Karussis et al. [30] found that MSC therapy in multiple sclerosis patients, during a study lasting 25 months, had no observed serious side effects. Most of the studies demonstrated that MSC single transplantation is safe and does not stimulate an immune response [31]. Long-termly studies are required to find any side effects and approve safety. Finally, further studies are needed to confirm this combination efficacy and safety. In the current study, we showed the MSC-CM effect as a whole. Thus, we recommend studying the special vital components that control the antifibrotic efficacy.

## 5. Conclusion

Finally, we conclude that Nilotinib synergized with liver stem cell conditioned media to achieve a safer and antifibrotic effect on activated, but not quiescent, HSCs than Nilotinib alone. Furthermore, long-termly toxicity examinations are needed to detect any harmful impact concerning the Nilotinib + liver MSC combination.

## Figures and Tables

**Table 1:** IC_50_ and SI calculations.

Compounds	Activated rat HSCs	Liver MSC	SI	WI-38	SI	MDBK	SI	CHO-K1	SI
DMSO	—	—	—	—	—	—	—	—	—
Nilotinib	25	40	1.6	35	1.4	40	1.6	15	0.6
CM + Nilotinib	10	>50	>5	>50	>5	>50	>5	45	4.5

**Table 2:** Collagen expression, DNA content, and ROS in HSCs. The values were expressed as M ± SD, and the effect size statistic was assessed by partial eta squared in each group.

Group parameter		Control	Nilotinib	Nilotinib + MSC-CM	Partial *η*^2^	Sig.
Collagen expression (OD)	Mean ± SEM	0.80 ± 0.12	0.57 ± 0.12^∗^	0.34 ± 0.08^∗^, #	0.818	0.006
	*P*_1_		0.090	0.005		
	*P*_2_			0.090		
DNA content *μ*g/100 mL cell lysate	Mean ± SEM	508.9 ± 67.5	340.1 ± 76.6^∗^	244.2 ± 61.4^∗^, #	0.791	0.009
	*P*_1_		0.054	0.008		
	*P*_2_			0.278		
ROS (OD)	Mean ± SEM	0.18 ± 0.077	0.37 ± 0.23^∗^	0.78 ± 0.09^∗^, #	0.808	0.007
	*P*_1_		0.332	0.006		
	*P*_2_			0.035		

*P*: probability. ^∗^Significant compared to the control group. #Significant compared to the Nilotinib group. *P*_1_ value for the control group. *P*_2_ value for the Nilotinib group. *η*^2^: eta squared. Test: one-way ANOVA, post hoc Tukey, and effect size statistic were assessed by partial eta squared.

## Data Availability

The data used to support the findings of this study are available from the corresponding author upon request

## Supplementary Materials

Supplementary Materials Supplementary Materials provide the original uncropped and unadjusted images used to create this article, western blotting panels showing the full background, loading wells, and size markers for 3 independent experiments. Figure S1: western blotting of cytosolic cytochrome c. Figure S2: western blotting of mitochondrial cytochrome c. Figure S3: western blotting of P53. Figure S4: western blotting of *α*-SMA. Figure S5: western blotting of cytosolic *β*-actin. Figure S6: western blotting of mitochondrial *β*-actin.

## References

[1] Brenner D. A. (2009). Molecular pathogenesis of liver fibrosis. *Transactions of the American Clinical and Climatological Association*.

[2] Kisseleva T., Brenner D. A. (2011). Anti-fibrogenic strategies and the regression of fibrosis. *Best Practice & Research Clinical Gastroenterology*.

[3] Nabil A., Elshemy M. M., Asem M. (2020). Zinc oxide nanoparticle synergizes sorafenib anticancer efficacy with minimizing its cytotoxicity. *Oxidative Medicine and Cellular Longevity*.

[4] Shiha G. E., Abu-Elsaad N. M., Zalata K. R., Ibrahim T. M. (2014). Tracking anti-fibrotic pathways of nilotinib and imatinib in experimentally induced liver fibrosis: an insight. *Clinical and Experimental Pharmacology & Physiology*.

[5] Berardis S., Sattwika P. D., Najimi M., Sokal E. M. (2015). Use of mesenchymal stem cells to treat liver fibrosis: current situation and future prospects. *World Journal of Gastroenterology*.

[6] Meier R. P. H., Mahou R., Morel P. (2015). Microencapsulated human mesenchymal stem cells decrease liver fibrosis in mice. *Journal of Hepatology*.

[7] Hu C., Zhao L., Duan J., Li L. (2019). Strategies to improve the efficiency of mesenchymal stem cell transplantation for reversal of liver fibrosis. *Journal of Cellular and Molecular Medicine*.

[8] Lee J. H., Park H. J., Kim Y. A. (2012). The phenotypic characteristic of liver-derived stem cells from adult human deceased donor liver. *Transplantation Proceedings*.

[9] Herrera M. B., Fonsato V., Bruno S. (2013). Human liver stem cells improve liver injury in a model of fulminant liver failure. *Hepatology*.

[10] Najimi M., Khuu D. N., Lysy P. A. (2007). Adult-derived human liver mesenchymal-like cells as a potential progenitor reservoir of hepatocytes?. *Cell Transplantation*.

[11] Shiha G., Nabil A., Lotfy A. (2020). Antifibrotic Effect of Combination of Nilotinib and Stem Cell-Conditioned Media on CCl4-Induced Liver Fibrosis. *Stem Cells International*.

[12] Aprigliano I., Dudas J., Ramadori G., Saile B. (2008). Atorvastatin induces apoptosis by a caspase-9-dependent pathway: an in vitro study on activated rat hepatic stellate cells. *Liver international*.

[13] Bhattacharya S., Prasanna A., Haldar P. K. (2011). Evaluation of antiproliferative activity of Trichosanthes dioica root against Ehrlich ascites carcinoma cells. *Academic Journal of Cancer Research*.

[14] Owen S. C., Doak A. K., Ganesh A. N. (2014). Colloidal drug formulations can explain “bell-shaped” concentration-response curves. *ACS Chemical Biology*.

[15] Weiskirchen R., Gressner A. M. (2005). Isolation and culture of hepatic stellate cells. *Methods in Molecular Medicine*.

[16] Kawala R. A., Ramadhani F. J., Choi H. J. (2019). Kenalog modified by ionizing radiation induces intrinsic apoptosis mediated by elevated levels of reactive oxygen species in melanoma cancer. *Oncology Reports*.

[17] Labarca C., Paigen K. (1980). A simple, rapid, and sensitive DNA assay procedure. *Analytical Biochemistry*.

[18] Gabele E., Brenner D. A., Rippe R. A. (2003). Liver fibrosis: signals leading to the amplification of the fibrogenic hepatic stellate cell. *Frontiers in Bioscience*.

[19] Issa R., Williams E., Trim N. (2001). Apoptosis of hepatic stellate cells: involvement in resolution of biliary fibrosis and regulation by soluble growth factors. *Gut*.

[20] Valenzuela M. T., Guerrero R., Núñez M. I. (2002). PARP-1 modifies the effectiveness of p53-mediated DNA damage response. *Oncogene*.

[21] Akhmetshina A., Dees C., Pileckyte M. (2008). Dual inhibition of c-abl and PDGF receptor signaling by dasatinib and nilotinib for the treatment of dermal fibrosis. *FASEB journal*.

[22] Day E., Waters B., Spiegel K. (2008). Inhibition of collagen-induced discoidin domain receptor 1 and 2 activation by imatinib, nilotinib and dasatinib. *European Journal of Pharmacology*.

[23] Liu Y., Wang Z., Kwong S. Q. (2011). Inhibition of PDGF, TGF-*β*, and Abl signaling and reduction of liver fibrosis by the small molecule Bcr-Abl tyrosine kinase antagonist Nilotinib. *Journal of Hepatology*.

[24] Shaker M. E., Shiha G. E., Ibrahim T. M. (2011). Comparison of early treatment with low doses of nilotinib, imatinib and a clinically relevant dose of silymarin in thioacetamide-induced liver fibrosis. *European Journal of Pharmacology*.

[25] Yin S., Ji C., Wu P., Jin C., Qian H. (2019). Human umbilical cord mesenchymal stem cells and exosomes: bioactive ways of tissue injury repair. *American Journal of Translational Research*.

[26] Friedman S. L. (2008). Hepatic stellate cells: protean, multifunctional, and enigmatic cells of the liver. *Physiological Reviews*.

[27] Chen S., Xu L., Lin N., Pan W., Hu K., Xu R. (2011). Activation of Notch1 signaling by marrow-derived mesenchymal stem cells through cell-cell contact inhibits proliferation of hepatic stellate cells. *Life Sciences*.

[28] Van Poll D., Parekkadan B., Cho C. H. (2008). Mesenchymal stem cell–derived molecules directly modulate hepatocellular death and regeneration in vitro and in vivo. *Hepatology*.

[29] Lalu M. M., McIntyre L., Pugliese C. (2012). Safety of cell therapy with mesenchymal stromal cells (SafeCell): a systematic review and meta-analysis of clinical trials. *PLoS One*.

[30] Karussis D., Karageorgiou C., Vaknin-Dembinsky A. (2010). Safety and immunological effects of mesenchymal stem cell transplantation in patients with multiple sclerosis and amyotrophic lateral sclerosis. *Archives of Neurology*.

[31] Cho P. S., Messina D. J., Hirsh E. L. (2008). Immunogenicity of umbilical cord tissue–derived cells. *Blood*.

