# Supplementary material for: The Potential Safe Antifibrotic Effect of Stem Cell Conditioned Medium and Nilotinib Combined Therapy by Selective Elimination of Rat Activated HSCs
Source: Biomed Res Int. 2021 Mar 28;2021:6678913. doi: 10.1155/2021/6678913 (PMC8021473; doi:10.1155/2021/6678913)
Supplement: Supplementary Materials — provide the original uncropped and unadjusted images used to create this article, western blotting panels showing the full background, loading wells, and size markers for 3 independent experiments. Figure S1: western blotting of cytosolic cytochrome c. Figure S2: western blotting of mitochondrial cytochrome c. Figure S3: western blotting of P53. Figure S4: western blotting of α-SMA. Figure S5: western blotting of cytosolic β-actin. Figure S6: western blotting of mitochondrial β-actin. [file 6678913.f1.docx]

**Supplementary Data**

**Figure S1: Western blotting of Cytosolic Cytochrome-c**

| 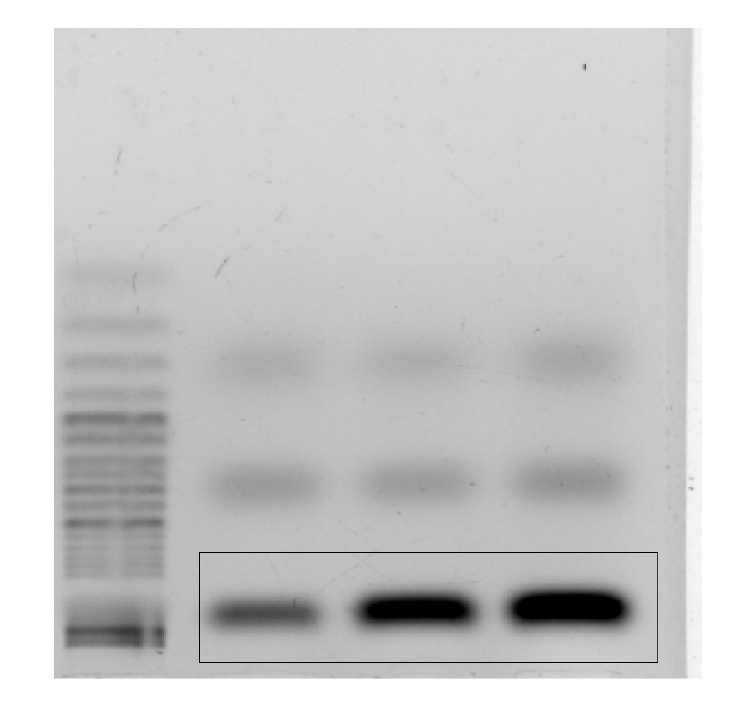  **A**  **CONTROL** **Nilotinib Nilotinib + MSC-CM** | 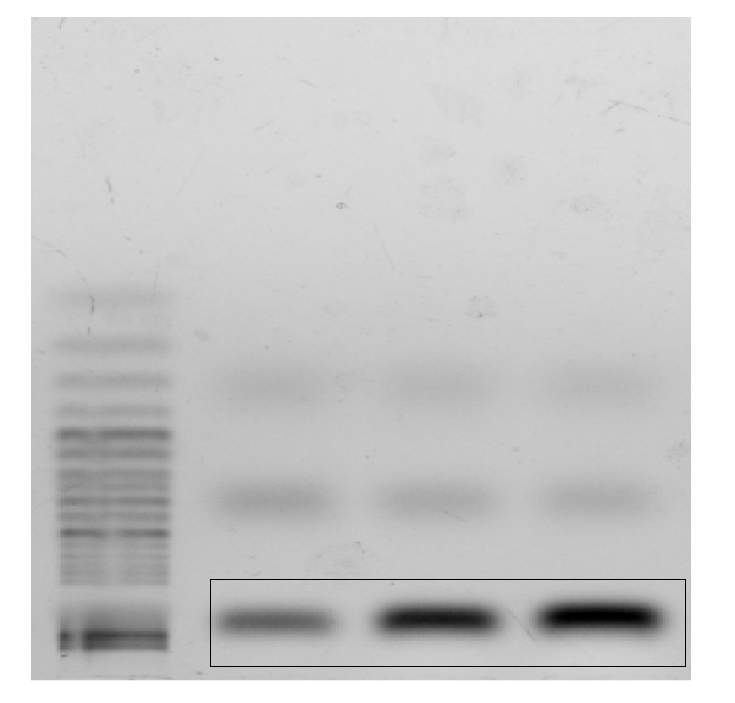  **B**  **CONTROL** **Nilotinib Nilotinib + MSC-CM** |
| --- | --- |
| 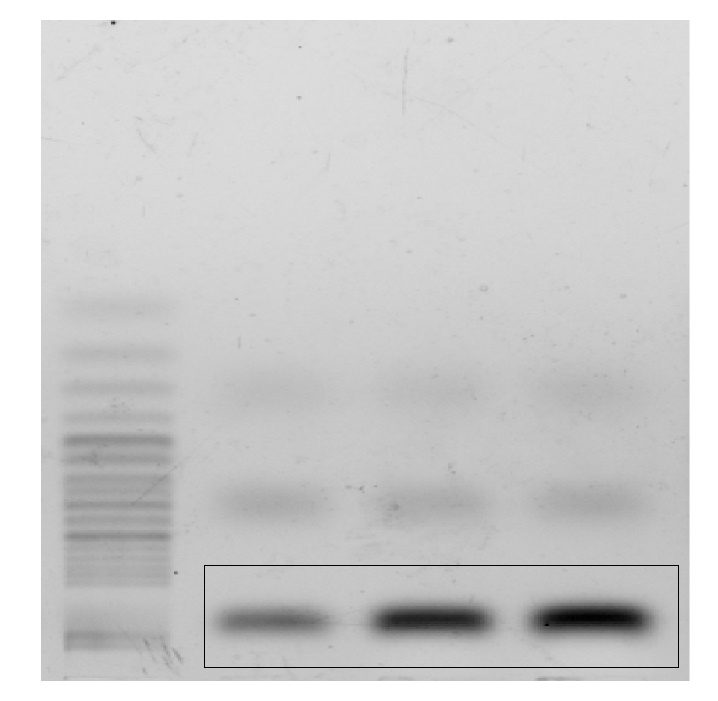  **C**  **CONTROL** **Nilotinib Nilotinib + MSC-CM** |  |

Figure S1. Western blotting assays were carried out to detect the effect of Nilotinib and MSC-CM on cytosolic cytochrome-c expression in HSCs. Results were obtained from 3 independent experiments first sample (Figure S1A), second sample (Figure S1B) and third sample (Figure S1C).

**Figure S2: Western blotting of Mitochondrial Cytochrome-c**

| 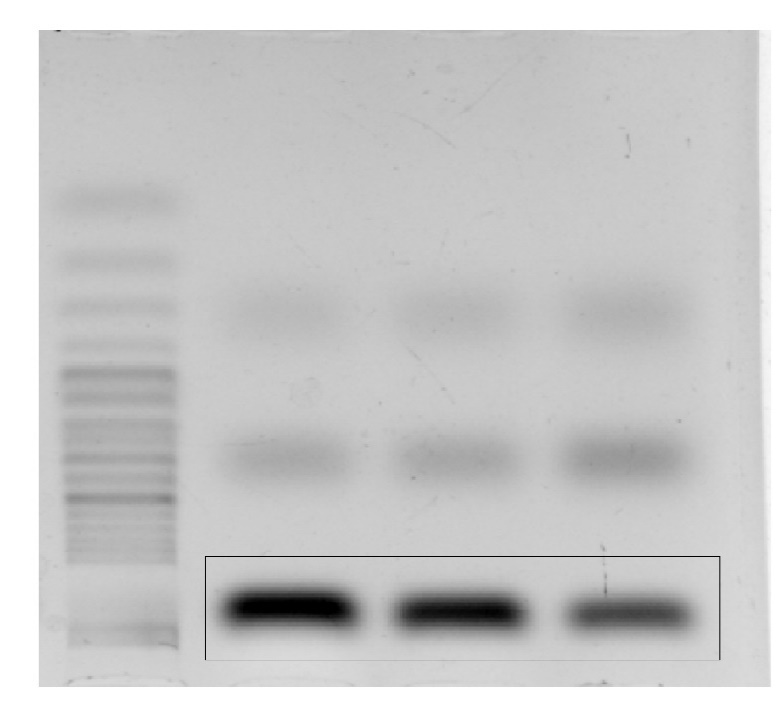  **A**  **CONTROL** **Nilotinib Nilotinib + MSC-CM** | 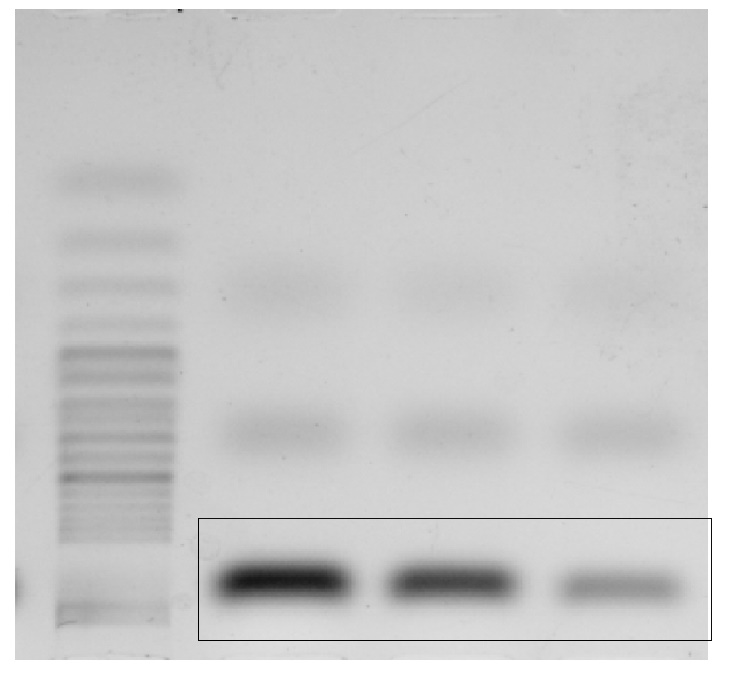  **B**  **CONTROL** **Nilotinib Nilotinib + MSC-CM** |
| --- | --- |
| 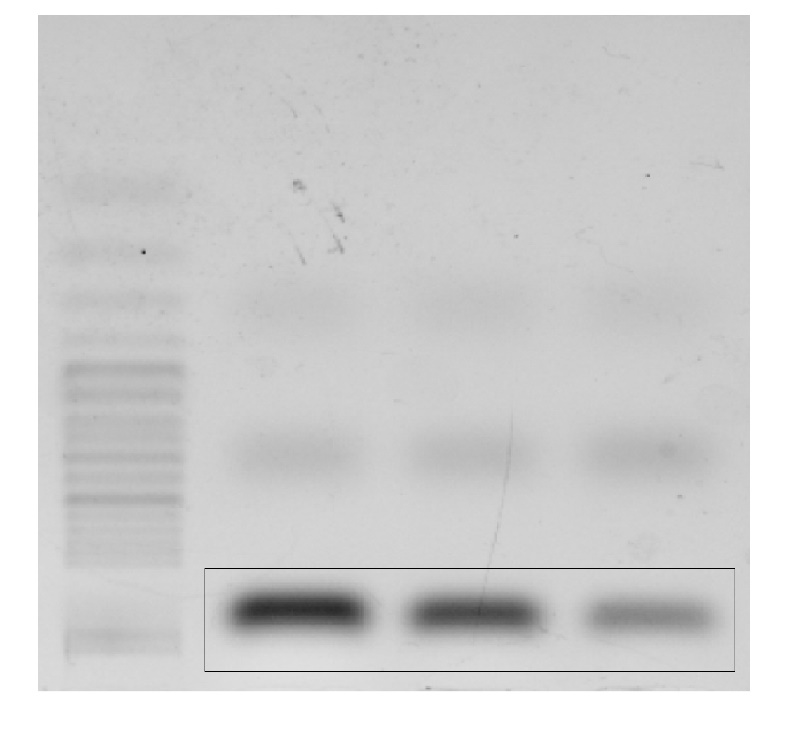  **C**  **CONTROL** **Nilotinib Nilotinib + MSC-CM** |  |

Figure S2. Western blotting assays were carried out to detect the effect of Nilotinib and MSC-CM on mitochondrial cytochrome-c expression in HSCs. Results were obtained from 3 independent experiments first sample (Figure S2A), second sample (Figure S2B) and third sample (Figure S2C).

**Figure S3: Western blotting of** **P53**

| 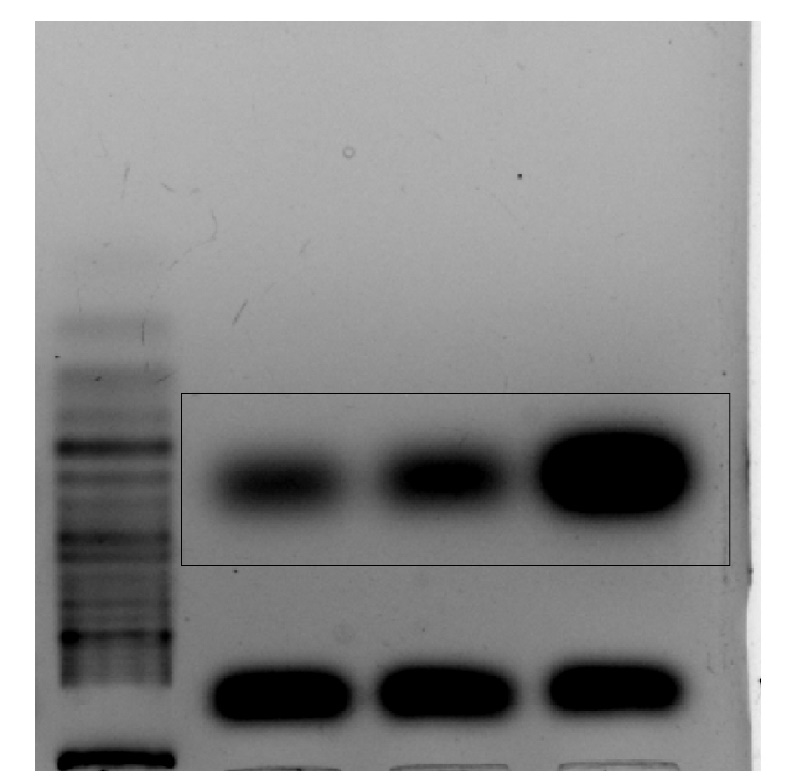  **A**  **CONTROL** **Nilotinib Nilotinib + MSC-CM** | 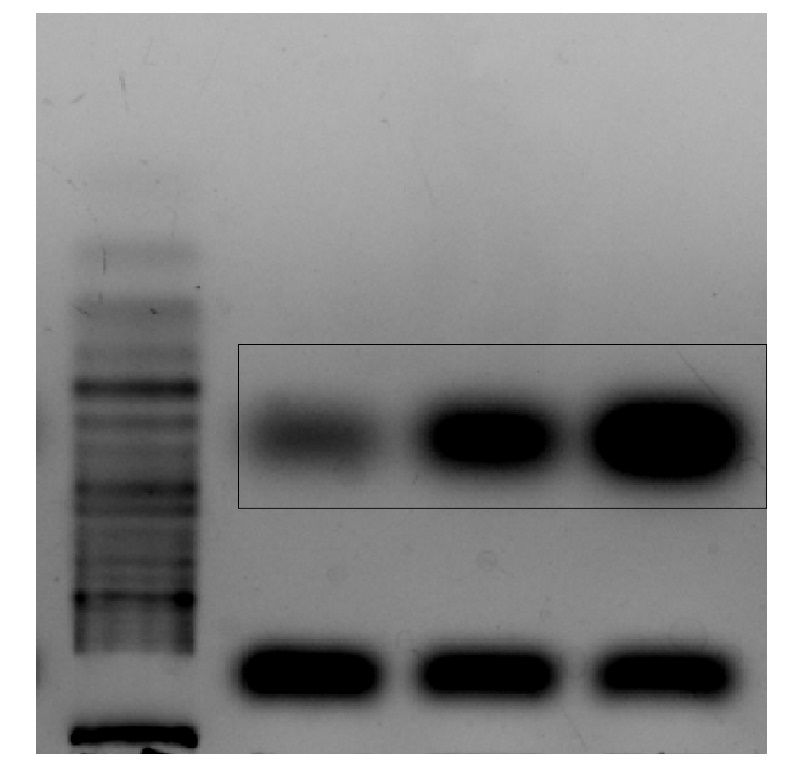  **B**  **CONTROL** **Nilotinib Nilotinib + MSC-CM** |
| --- | --- |
| 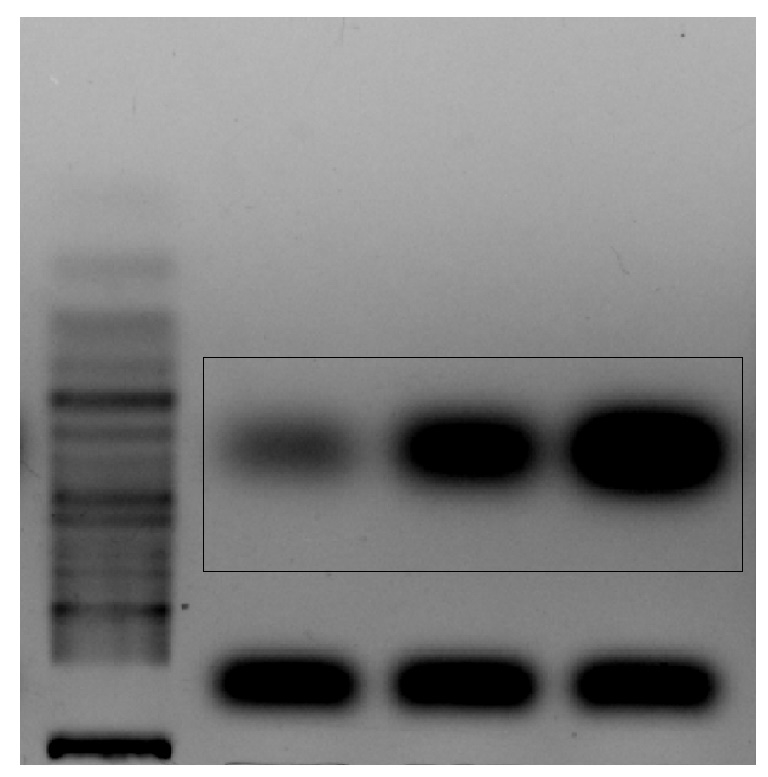  **C**  **CONTROL** **Nilotinib Nilotinib + MSC-CM** |  |

Figure S3. Western blotting assays were carried out to detect the effect of Nilotinib and MSC-CM on P53 expression in HSCs. Results were obtained from 3 independent experiments first sample (Figure S3A), second sample (Figure S3B) and third sample (Figure S3C).

**Figure S4: Western blotting of α-SMA**

| 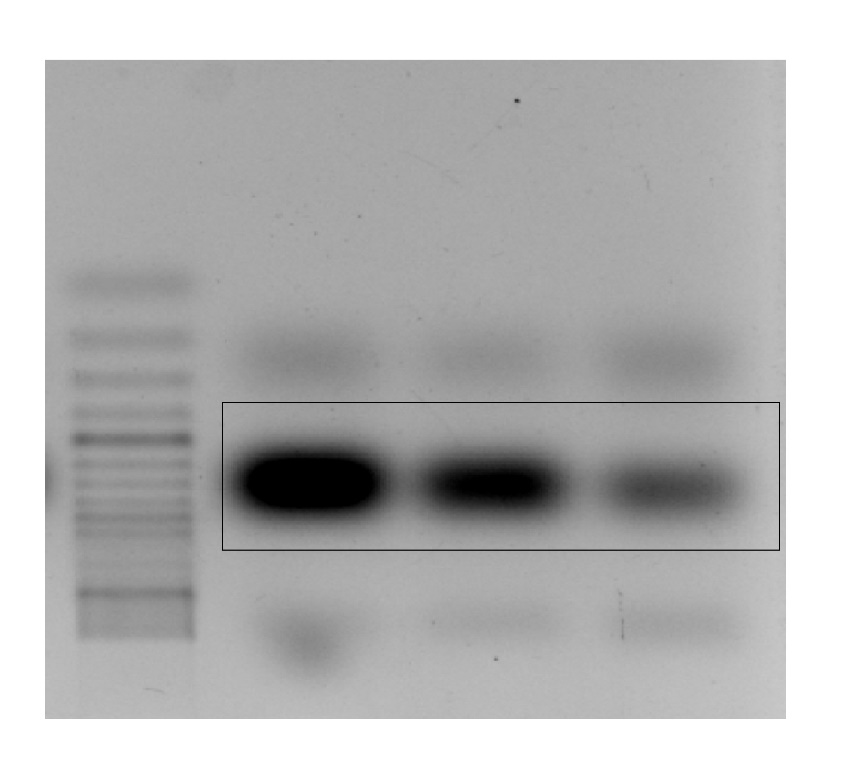  **A**  **CONTROL** **Nilotinib Nilotinib + MSC-CM** | 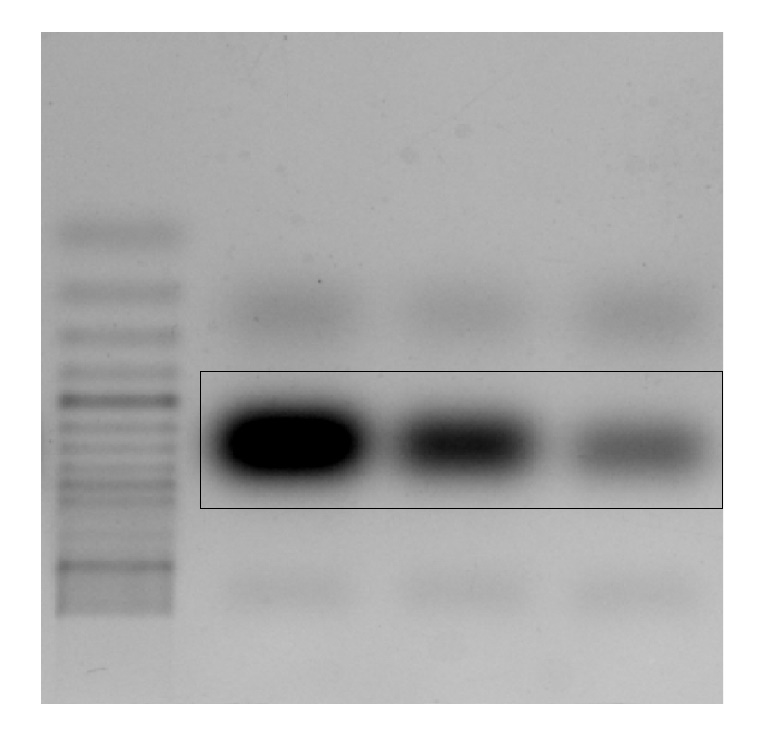  **B**  **CONTROL** **Nilotinib Nilotinib + MSC-CM** |
| --- | --- |
| **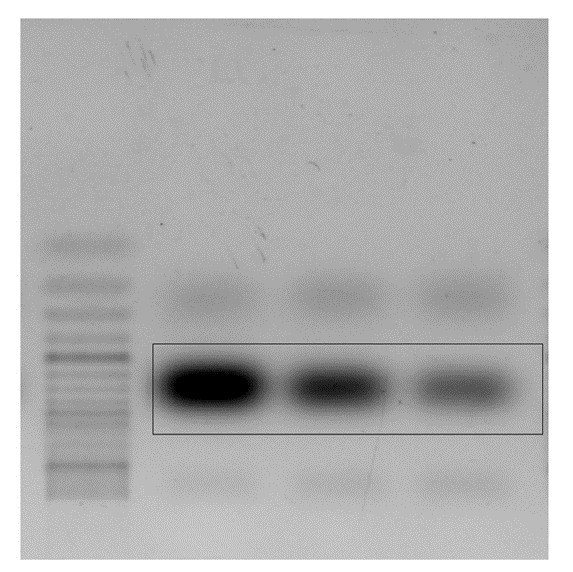**  **C**  **CONTROL** **Nilotinib Nilotinib + MSC-CM** |  |

Figure S4. Western blotting assays were carried out to detect the effect of Nilotinib and MSC-CM on α-SMA expression in HSCs. Results were obtained from 3 independent experiments first sample (Figure S4A), second sample (Figure S4B) and third sample (Figure S4C).

**Figure S5: Western blotting of** **Cytosolic β-ACTIN**

| 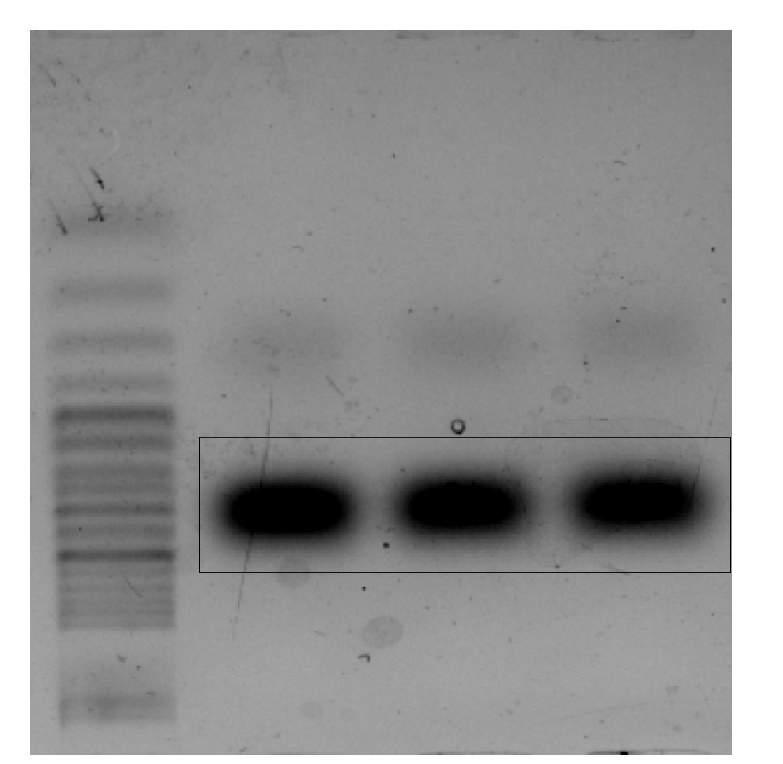  **A**  **CONTROL** **Nilotinib Nilotinib + MSC-CM** | 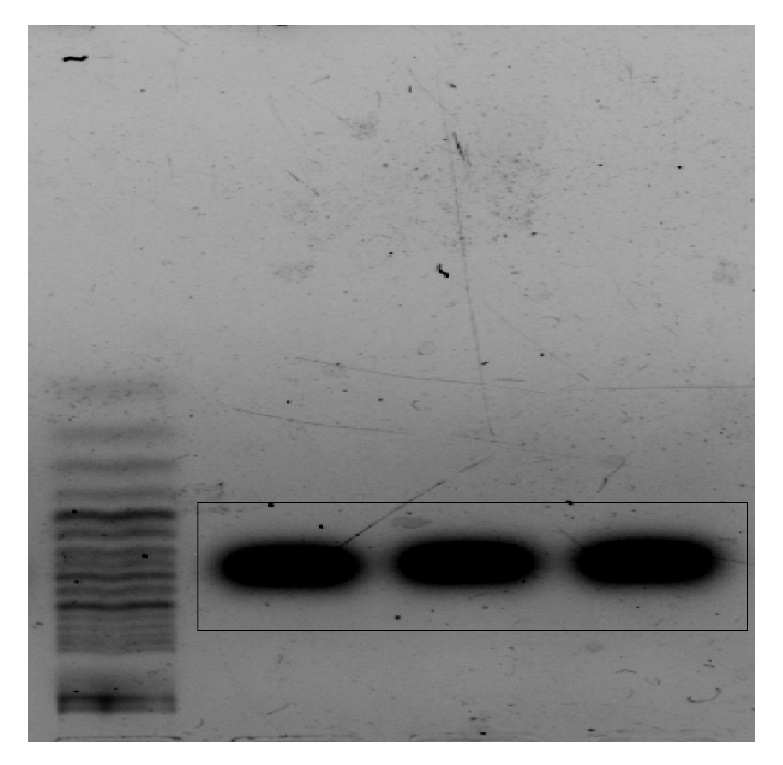  **B**  **CONTROL** **Nilotinib Nilotinib + MSC-CM** |
| --- | --- |
| 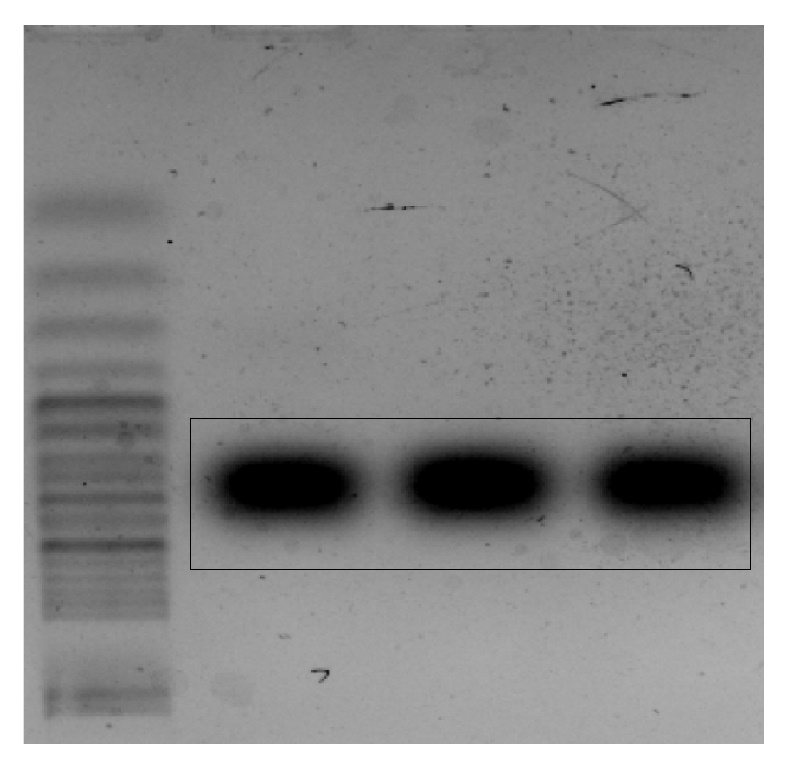  **C**  **CONTROL** **Nilotinib Nilotinib + MSC-CM** |  |

Figure S5. Western blotting assays were carried out to detect the effect of Nilotinib and MSC-CM on cytosolic β-ACTIN expression in HSCs. Results were obtained from 3 independent experiments first sample (Figure S5A), second sample (Figure S5B) and third sample (Figure S5C).

**Figure S6: Western blotting of Mitochondrial β-ACTIN**

| 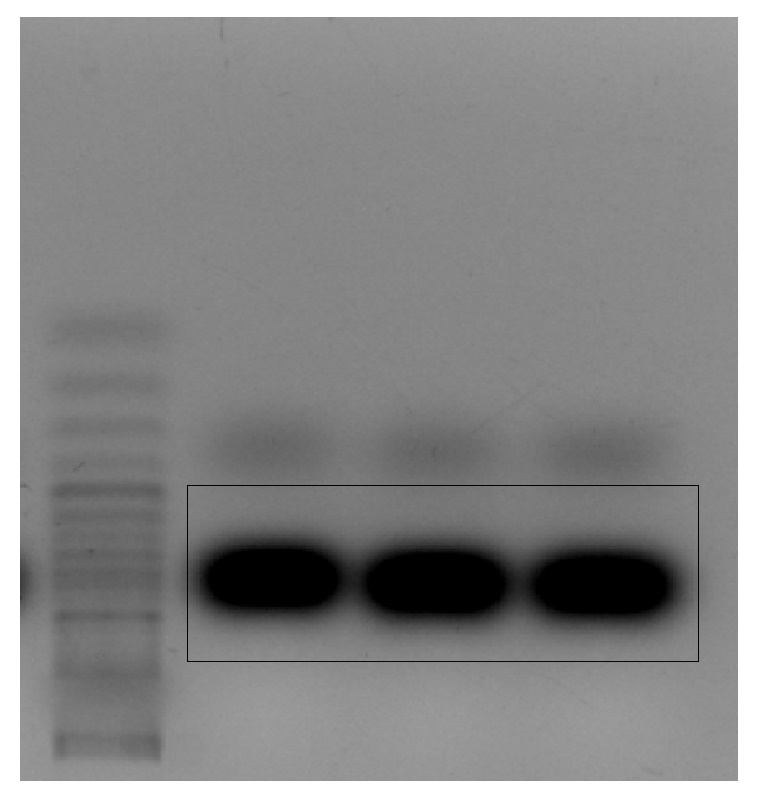  **A**  **CONTROL** **Nilotinib Nilotinib + MSC-CM** | 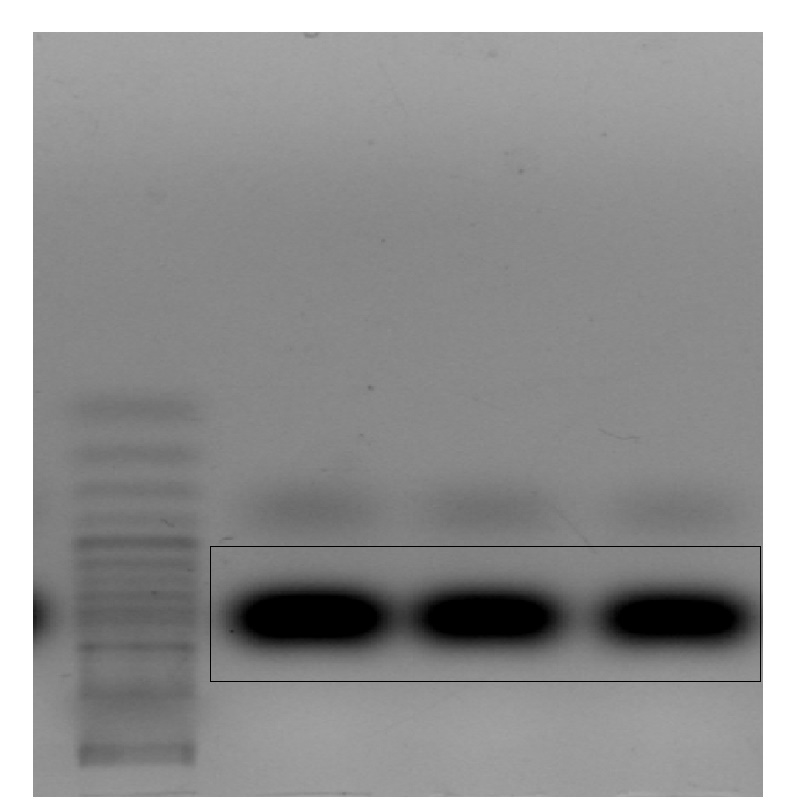  **B**  **CONTROL** **Nilotinib Nilotinib + MSC-CM** |
| --- | --- |
| 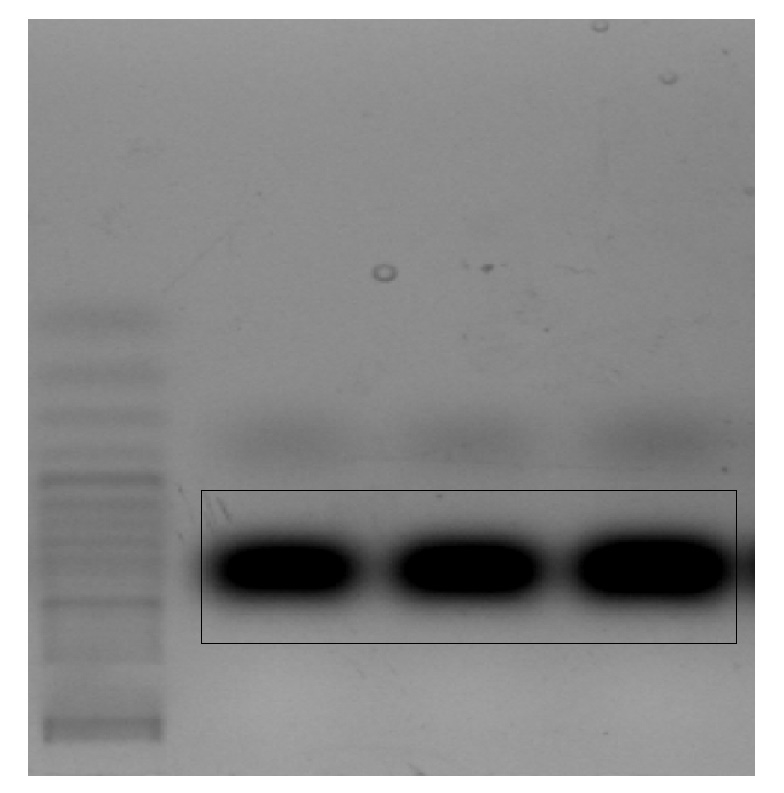  **C**  **CONTROL** **Nilotinib Nilotinib + MSC-CM** |  |

Figure S6. Western blotting assays were carried out to detect the effect of Nilotinib and MSC-CM on mitochondrial β-ACTIN expression in HSCs. Results were obtained from 3 independent experiments first sample (Figure S6A), second sample (Figure S6B) and third sample (Figure S6C).
